# Analysis of ROMO1 Expression Levels and Its Oncogenic Role in Gastrointestinal Tract Cancers

**DOI:** 10.3390/cimb46120863

**Published:** 2024-12-20

**Authors:** Selçuk Yaman, Osman Akidan, Mehmet Vatansever, Sema Misir, Serap Ozer Yaman

**Affiliations:** 1 Department of Medical Biochemistry, Trabzon Kanuni Training and Research Hospital, Trabzon 61250, Turkey; dr_selcukyaman@hotmail.com; 2 Department of Hematology, Mengücek Gazi Education and Research Hospital, Erzincan 24100, Turkey; 3 Department of Family Medicine, Trabzon Kanuni Training and Research Hospital, Trabzon 61250, Turkey; 4 Department of Biochemistry, Faculty of Pharmacy, Sivas Cumhuriyet University, Sivas 58140, Turkey; 5 Department of Medical Biochemistry, Trabzon Kanuni Health Practice and Research Hospital, Trabzon Faculty of Medicine, University of Health Sciences, Trabzon 61250, Turkey

**Keywords:** ROMO1, gastrointestinal cancers, immune cells

## Abstract

Gastrointestinal tract cancers account for approximately one-third of cancer-related deaths. Early diagnosis and effective treatment are the most important ways to prevent cancer-related morbidity and mortality. ROMO1 has been shown to play an important role in many types of cancer. However, the biological function of ROMO1 is still poorly understood in gastrointestinal system cancers. The aim of this study is to reveal the expression change and oncogenic role of ROMO in gastrointestinal system cancers. Gene Expression Profiling Interactive Analysis (GEPIA), UALCAN, TIMER, GeneMANIA, TISIDB, and STRING were applied to assess the biological function of ROMO1 in gastrointestinal cancers (colon adenocarcinoma (COAD), esophageal carcinoma (ESCA), liver hepatocellular carcinoma (LIHC), pancreatic adenocarcinoma (PAAD), and stomach adenocarcinoma (STAD)). ROMO1 is significantly increased in COAD, ESCA, LUHC, and PAAD, and the overexpression of ROMO1 is associated with clinicopathological features. In addition, ROMO1 has been found to be closely associated with tumor-infiltrating immune cells in gastrointestinal cancers. ROMO1 is closely related to the inner mitochondrial membrane proteins (TIMM) family. The study revealed that ROMO1 is of significant clinical importance for gastrointestinal cancers and may have potential clinical utility in treatment and prognosis. Functional tests on cell lines derived from these particular gastrointestinal cancers can also be performed in vitro to evaluate the impact of the ROMO1 gene and other factors, like potential drugs, on the expression of these genes and the development and progression of the cancer.

## 1. Introduction

Cancer is a heterogeneous disease characterized by processes such as the excessive proliferation of cells, dysfunction of cell death mechanisms, invasion, and metastasis [1]. Today, it is one of the leading health problems due to its incidence and mortality rate [2]. Gastrointestinal (GI) tract cancers are a group of cancers of the GI tract and digestive organs, such as the stomach, heart, bile ducts, pancreas, esophagus, colon, and rectum [3]. GI cancers account for almost one-third of cancer-related deaths. It has been reported that GI cancers accounted for approximately 26% of the total cancer incidence and approximately 35% of all cancer-related deaths in 2018 [4]. GI cancer is estimated to reach 7.5 million new cases and 5.6 million deaths by 2040 [5]. Despite advances in technology and new treatment strategies, GI cancers remain a significant global burden to human health [6,7]. Therefore, it is essential to study the development of GI cancers at the molecular level, identify the genetic factors that play a role in their pathogenesis, and improve our understanding of their early diagnosis and treatment.

Reactive oxygen species modulator 1 (ROMO1) is a mitochondrial inner membrane channel protein that plays a role in reactive oxygen species (ROS) production and regulation [8,9]. It also plays a critical role in maintaining the morphology of mitochondria and the integrity of their inner membrane structures [9,10]. ROS and oxidative stress are the most important causes that play a role in the development and progression of cancer [8,11]. ROMO1, a protein that regulates ROS production, has been shown to play an essential role in many types of cancer [8,12]. ROS can lead to proliferation and metastasis in various types of cancer through different pathways, including nuclear factor kappa B (NF-κB), extracellular-signal-regulated kinase (ERK), mitogen-activated protein kinase (MAPK), tumor protein p53 (p53), and phosphoinositide 3-kinases (PI3-K) [9,13,14,15]. For example, ROMO1 has been associated with poor prognosis in colorectal cancer patients [13], and poor survival in non-small cell lung cancer (NSCLC) patients [16]. ROMO1 has been indicated to regulate ROS production and cell growth in gliomas [17].

In clinical laboratories, a number of tumor markers are used to diagnose and identify a neoplasm. However, according to the information mentioned above, ROMO1 may have properties that can be used as a tumor marker in diagnostic laboratories and also as a treatment option. Since ROMO1 is associated with the level of oxidative stress and ROS production in cancer cells, and by regulating its expression, it can reduce cancer symptoms and better response to chemotherapy [11]. Therefore, it is important to study the effects of ROMO1.

These days, bioinformatics analyses using large sample sizes and sophisticated algorithms are far more representative and dependable due to the quick development of biological databases. The use of bioinformatic analysis to analyze the course of cancer and pinpoint prospective treatment targets has increased significantly in recent years [18]. These investigations have the advantage of being able to overcome conflicting results seen in the literature because of their various sample sizes, microarray technology, and sequencing platforms [18].

A thorough comprehension of the molecular mechanism underlying GI cancer pathogenesis is probably going to offer justification for creating and constructing a suitable treatment. Herein, we used a variety of bioinformatics techniques to investigate ROMO1 expression profiles, diagnostic value, genetic alteration, protein methylation level, immunological infiltration, and functional states in GI cancers. In order to ascertain their significance in the pathophysiology of neoplastic alterations in the GI tract, the current study subjected them to bioinformatics analysis because the ROMO1 gene has such a powerful influence on numerous processes that determine autonomy and escape from immune regulation. To the best of our knowledge, this is the first thorough investigation to assess the involvement of the whole ROMO1 gene in a subset of gastrointestinal malignancies. This comprehensive analysis revealed the oncogenic role of ROMO1 in gastrointestinal tract cancers, the potential value of ROMO1 in the diagnosis of GI cancers, the underlying molecular mechanisms of ROMO1 in GI cancer pathogenesis, and the effects of ROMO1 in anti-tumor immune response and therapeutic target in the treatment of GI cancers.

## 2. Materials and Methods

### 2.1. Expression Analysis of ROMO1

The “Diff Exp” module of the Tumor Immunity Evaluation Resource (TIMER) website (http://timer.cistrome.org/, accessed on 15 August 2024) was used to investigate various primary tumor types and normal control tissues in The Cancer Genome Atlas Program (TCGA) database [19]. Gene Expression Profiling Interactive Analysis, version 2 (GEPIA2) l (http://gepia2.cancer-pku.cn/#analysis, accessed on 19 August 2024), and University of Alabama at Birmingham Cancer data analysis Portal (UALCAN) tools (http://ualcan.path.uab.edu/, accessed on 19 August 2024) were used to analyze the gene expression of ROMO1 in gastrointestinal cancers (colon adenocarcinoma (COAD), esophageal carcinoma (ESCA), liver hepatocellular carcinoma (LIHC), pancreatic adenocarcinoma (PAAD), and stomach adenocarcinoma (STAD)). Using the gene expression profiling Gene Expression Profiling Interactive Analysis (GEPIA) (http://gepia.cancer-pku.cn/, accessed on 19 August 2024) database, we identified changes in the expression of identified genes between GI cancers and healthy tissue [20]. UALCAN is a fast and effective online analysis and mining website, mainly based on the TCGA database cancer data, and can provide a large number of comprehensive analyses, including gene expression, survival analysis, and epigenetic regulation [21].

### 2.2. Survival Analysis of ROMO1

Overall survival (OS) map data of ROMO1 for COAD, ESCA, LIHC, PAAD, and STAD were obtained from the “Survival Map” module in GEPIA with a 50% cut-off value to separate groups into high expression and low expression, and disease-free survival (DFS) was determined [20]. UALCAN has made it possible for users to assess the expression of protein-coding genes and how it affects patient survival in 33 different cancer types. Additionally, the UALCAN online tool was used to perform survival analysis of ROMO1 in the same cancer types [21].

### 2.3. ROMO1 Expression in Molecular and Immune Subtypes of GI Cancers

The Tumor-Immune System Interaction Database (TISIDB) is an online platform that integrates many heterogeneous data sources for tumor and immune system interaction. The TISIDB (http://cis.hku.hk/TISIDB/, accessed on 30 August 2024), a comprehensive database, can be used to examine how neoplasms and the immune system interact [22]. The TISIDB was used to analyze the association between ROMO1 expression and molecular or immune subtypes in COAD, ESCA, LIHC, PAAD, and STAD.

### 2.4. DNA Methylation Analysis

Methylation level of ROMO1 in GI cancers and corresponding normal tissues was investigated in the UALCAN database [21]. In this database, you can set the conditions for filtering and data mining. The screening conditions set in this study are gene: ROMO1; cancer type: COAD, ESCA, LIHC, PAAD, and STAD; data type: TCGA dataset. The Beta value indicates the level of DNA methylation ranging from 0 (unmethylated) to 1 (fully methylated).

### 2.5. Correlation Between ROMO1 and Immune Infiltration

The Tumor Immunity Evaluation Resource is a visualization website that can analyze immune infiltration in various tumors [19]. The correlation between ROMO1 and the abundance of six immune infiltrates (B cells, CD4+ T cells, CD8+ T cells, neutrophils, macrophages, and dendritic cells) was evaluated. The TIMER 2.0 web server was used to investigate the correlation between ROMO1 and the infiltration of immune cells in COAD, ESCA, LIHC, PAAD, and STAD in TCGA. The findings were considered reliable when similar results were obtained using at least two algorithms. p-values were calculated by Spearman’s rank correlation test.

### 2.6. Analysis of the Gene and Protein That Interact with ROMO1 in Pan-Cancer

Search Tool for the Retrieval of Interacting Genes/Proteins (STRING) database (http://www.string-db.org/, accessed on 15 August 2024) was used to construct protein interaction networks of ROMO1 in cancer, and GeneMANIA (http://genemania.org/, accessed on 15 August 2024) was employed to analyze the interaction gene with the ROMO1. The STRING (http://www.string-db.org/) database is a protein–protein association network that provides all known and predicted information about the direct (physical) and indirect (functional) relationships that occur between different proteins [23]. Another online resource, GeneMANIA (http://genemania.org/) is a database consisting of an intuitive interface for gene function predictions and the interactions of genes with each other [24].

## 3. Results

**Figure 1:**
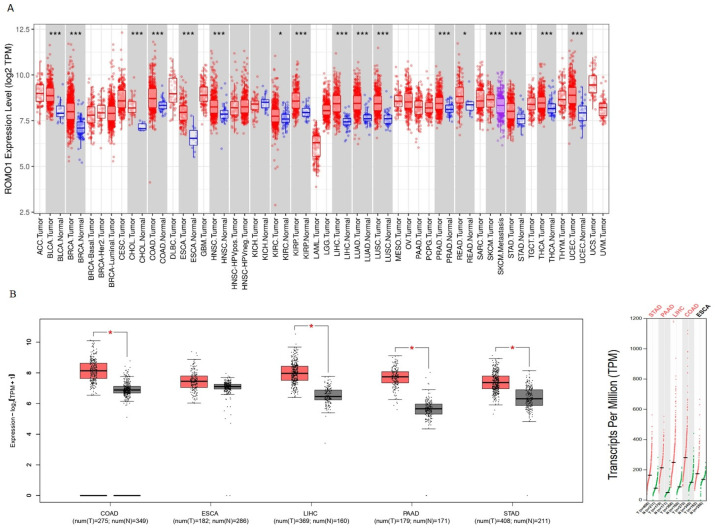

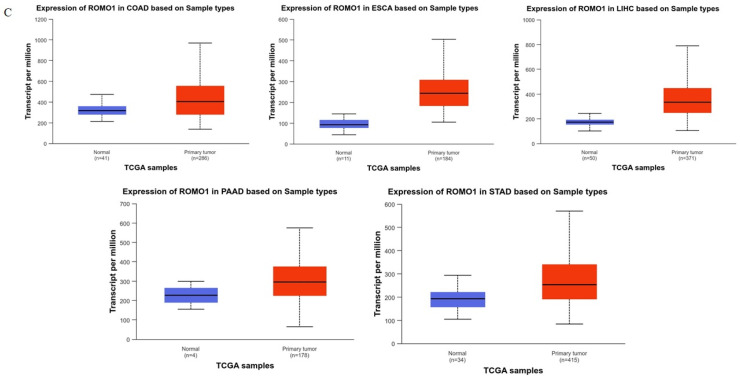
(**A**) The expression status of the ROMO1 gene in different cancer types was analyzed through TIMER2 (data from TCGA). * *p* < 0.05; *** *p* < 0.001. (**B**) The expression status of the ROMO1 gene in COAD, ESCA, LIHC, PAAD, and STAD was analyzed through GEPIA2 (data from TCGA and GTEx). * *p* < 0.05. (**C**) The expression of ROMO1 in COAD, ESCA, LIHC, PAAD, and STAD analysis using the UALCAN database.

**Figure 2:**
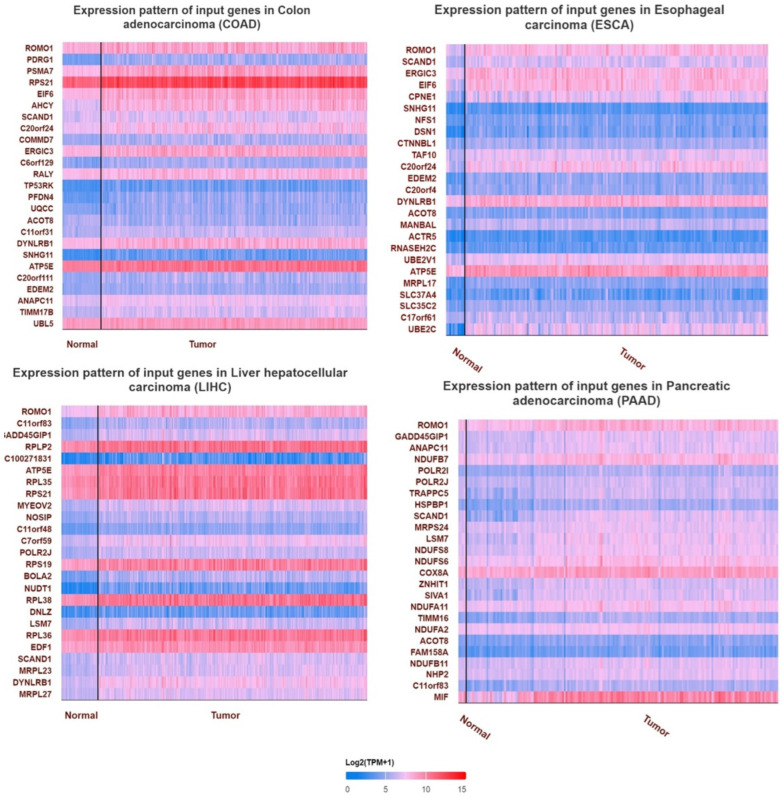
Heatmaps of significant ROMO1 in COAD, ESCA, LIHC, and PAAD.

Initially, we examined the mRNA and protein expression levels of ROMO1 in various GI cancers. We used the TIMER database to examine the expression of ROMO1 in different cancer types. ROMO1 was found to have high expression in COAD, ESCA, PAAD, LIHC, and STAD (Figure 1A). In addition, analysis of ROMO1 mRNA expression between normal tissues and cancers using GEPIA revealed that ROMO1 showed significantly higher expression in COAD, ESCA, PAAD, LIHC, and STAD (Figure 1B). Then, UALCAN was applied to determine the protein expression level of ROMO1. ROMO1 was also highly expressed in COAD, ESCA, PAAD, LIHC, and STAD (Figure 1C). Overall, these results indicate that ROMO1 expression is upregulated in gastrointestinal tract cancers. Moreover, a heat map image of ROMO1 and related genes in COAD, ESCA, LIHC, and PAAD is shown in Figure 2. In total, 25 genes positively correlated with ROMO1 were shown in COAD, ESCA, LIHC, and PAAD. These heatmaps were visualized as scatter plots using Pearson correlation list coefficient. Genes with extremely low expression (median TPM < 0.5) were filtered from the list.

**Figure 3:**
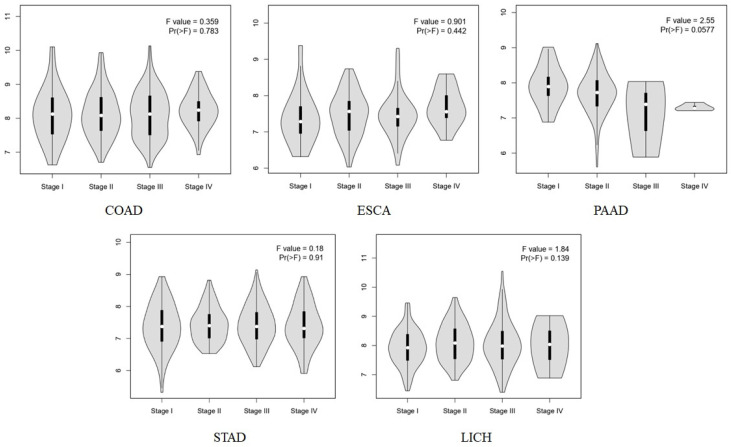
The expression levels of the ROMO1 gene were analyzed by the main pathological stages (stage I, stage II, stage III, and stage IV) of COAD, ESCA, LIHC, PAAD, and STAD.

To determine whether ROMO1 was differentially expressed among pathological stages, we first analyzed the correlations between mRNA expression of ROMO1 and pathological stages among gastrointestinal tumors using GEPIA2. The results showed that ROMO1 expression was not significantly associated with the pathological stage of gastrointestinal cancer types (*p* < 0.05, Figure 3).

**Figure 4:**
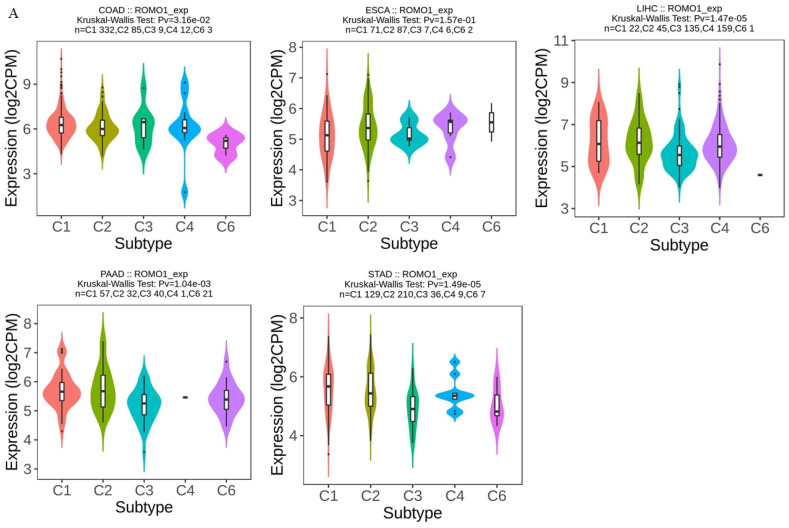

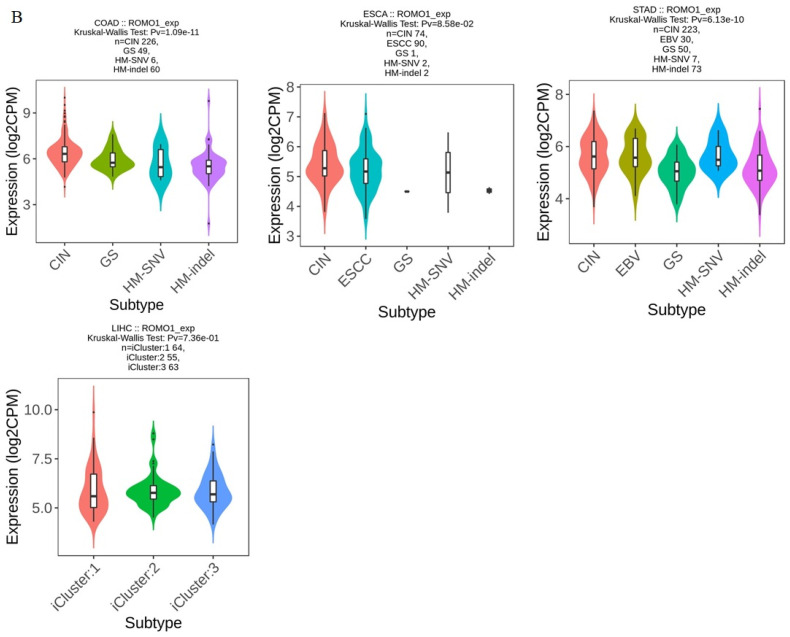
(**A**) Correlations between ROMO1 expression and immune subtypes in GI cancers. (**B**) Correlations between ROMO1 expression and molecular subtypes in GI cancers. Correlation analysis between ROMO1 expression, immune subtypes, and molecular subtypes was performed on the TISIDB.

Correlation analysis was performed between the expression of ROMO1 in intestinal cancers and molecular or immune subtypes from the TISIDB. For immune subtypes of GI cancers (C1: wound healing, C2: IFN-gamma dominant, C3: inflammatory, C4: lymphocyte depleted, C5: immunologically silent, C6: TGF-b dominant), ROMO1 expression was observed to be significantly different (Figure 4A). The results showed that ROMO1 was differentially expressed in GI cancers for molecular subtypes (Figure 4B).

**Figure 5:**
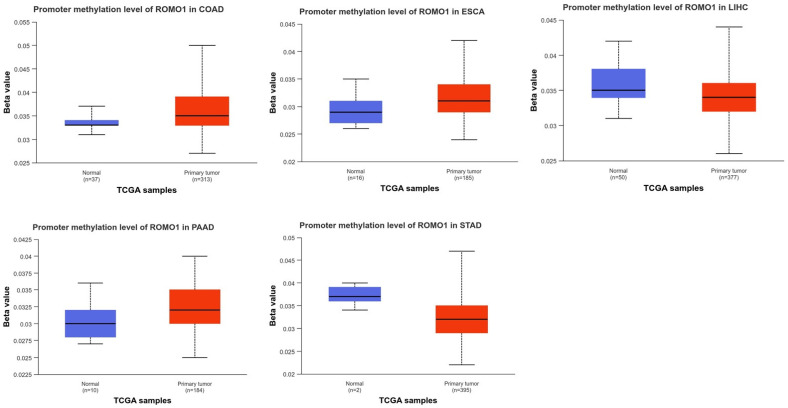
DNA methylation features of ROMO1 in GI cancers. The methylation level of ROMO1 obtained using UALCAN database.

It has been established that DNA methylation is crucial to the development and spread of malignancies. Furthermore, DNA methylation across the genome is an epigenetic alteration that helps control genes linked to cancer in a number of malignancies. The underlying roles of ROMO1 methylation in various cancers remain largely unclear. We evaluated the methylation level of ROMO1 in normal and GI cancers tissues using the UALCAN database. In our study, we demonstrated the decreased promoter methylation level of ROMO1 for LICH, STAD, and increased the promoter methylation level of ROMO1 for COAD, ESCA, and PAAD (Figure 5). Median values of ROMO1 promoter methylation level for normal and tumor tissue COAD (median: 0.033, 0.035), ESCA (median: 0.029, 0.031), LICH (median: 0.035, 0.034), PAAD (median: 0.03, 0.032), and STAD (median: 0.037, 0.032).

**Figure 6:**
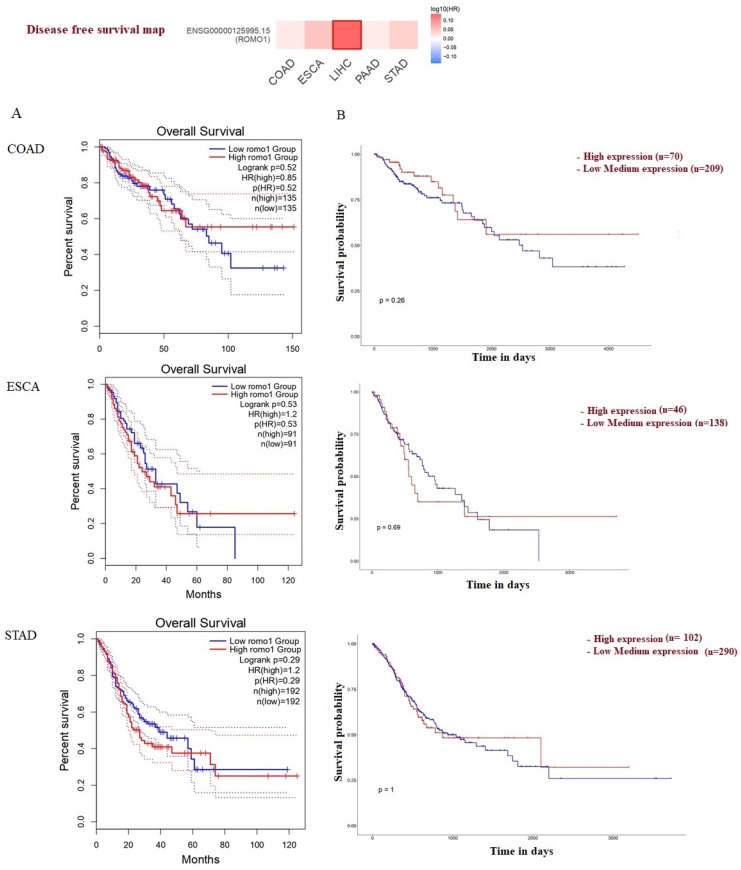

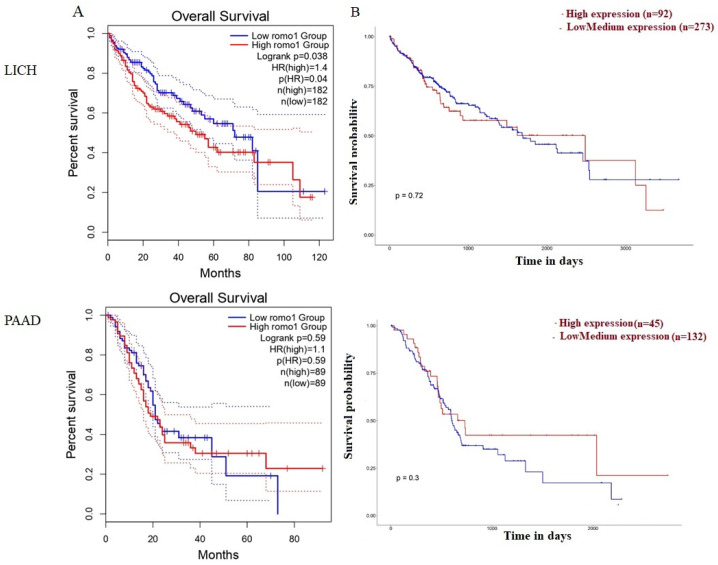
Disease-free survival for ROMO1. Analysis of the overall survival for ROMO1 in COAD, ESCA, LIHC, PAAD, and STAD using the GEPIA and ULACAN databases.

We used GEPIA and ULACAN to evaluate the survival and prognostic value of ROMO1 in COAD, ESCA, LICH, PAAD, and STAD. A high expression of ROMO1 was not related to poor OS in COAD, ESCA, PAAD, and STAD. A high expression of ROMO1 (*p* = 0.038) predicted poor OS, and high expression was associated with poor disease-free survival (DFS) in LICH (Figure 6). These results indicated the promising roles of ROMO1 in the patients’ prognosis of LICH.

**Figure 7:**
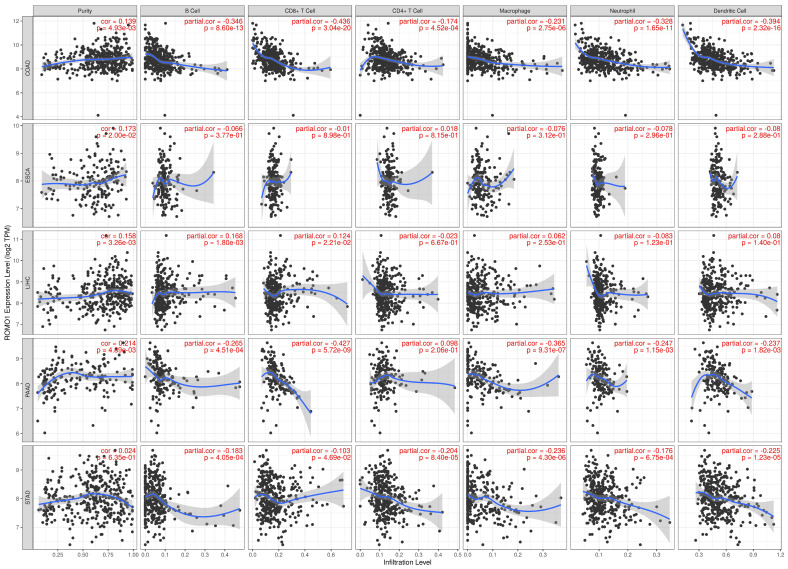
Association between ROMO1 and immune infiltration in COAD, ESCA, LIHC, PAAD and STAD using the TIMER.

The immune cell infiltration was obtained from the TIMER website. The expression level of ROMO1 was found to be strongly correlated with the abundance of six immune infiltrates (B cells, CD4+ T cells, CD8+ T cells, Neutrophils, Macrophages, and Dendritic cells) in COAD, LIHC, PAAD, and STAD (Figure 7).

**Figure 8:**
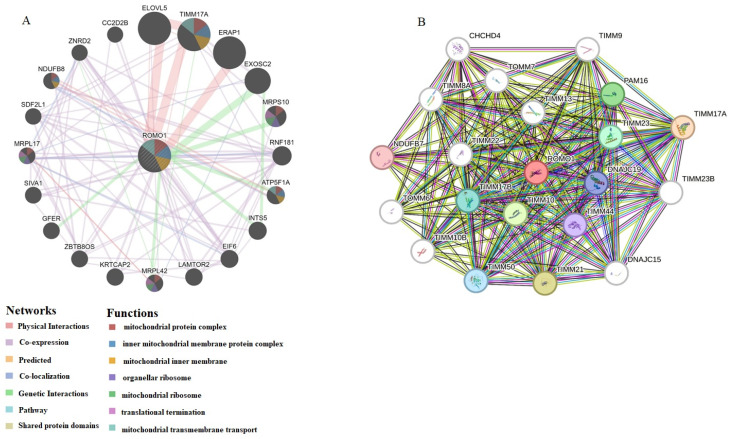
(**A**) The gene interaction network of ROMO1 was constructed using GeneMANIA. (**B**) The STRING database was employed to construct the protein interaction network of ROMO1.

Finally, to better understand the molecular mechanism of ROMO1 in tumorigenesis and development, we used the STRING and GeneMANIA tool to construct the ROMO1-interacted molecule network. To explore the regulatory mechanism of ROMO1, GeneMANIA was used to search for related genes (Figure 8A). We found that 20 main genes were associated with ROMO1, such as ELOVL5, TIMM17A, ERAP1, EXOSC2, MRPS10, RNF181, ATP5F1A, INTS5, EIF6, LAMTOR2, MRPL42, KRTCAP2, ZBTB8OS, GFER, SIVA1, MRPL17, SDF2L1, NDUFB8, ZNRD2 and CC2D2B. The proteins most closely associated with ROMO1 are TIMM23B, DNAJC15, TIMM17A, TIMM21, TIMM10, PAM16, TIMM23, TIMM17B, TIMM50, DNAJC19, TIMM44, NDUFB7, TIMM23B, DNAJC15, TIMM22, TIMM9, TIMM13, TIMM10B, TIMM8A, TOMM7, CHCHD4, and TOMM6 (Figure 8B). These proteins appear active in cell proliferation, cell cycle, and cell death pathways.

## 4. Discussion

One of the most prevalent types of cancer worldwide is GI tract malignant tumors. All can be impacted by lifestyle choices and living conditions, but many have also been shown to be strongly impacted by molecular alterations in genes and proteins [2]. A fundamental feature of all gastrointestinal cancers is the involvement of genomic and epigenomic alterations. Cancer cells further complicate the disease by altering the molecular and cellular biological processes of cells [1]. Intratumoral heterogeneity and the increasingly complex process of malignancies with disease progression continue to be the greatest obstacles to cancer treatment [25]. Therefore, early diagnosis and prognosis prediction for various malignancies are the most important ways to prevent cancer-related morbidity and mortality [2]. Although many anti-tumor treatments have been developed in recent years based on differences in tumor cell metabolism [26], further studies are needed to understand the mechanisms underlying cancer development and to provide more effective and new treatment strategies. This study aimed to investigate the clinical relevance of ROMO1 expression in GI cancers.

ROMO1 is a membrane protein in mitochondria that regulates mitochondrial ROS production, redox, mitochondrial dynamics, and apoptosis [27,28,29]. Increased ROS production alters intracellular oxidative stress homeostasis, can cause cell death, inflammation and persistent oxidative stress, enhance malignancy, and promote cancer development and progression [30]. Recent studies have implicated ROMO1 in various types of cancer, including hepatocellular carcinoma, colorectal, prostate, gastric, bladder, lung, and glioma [8,9,13,16,28,30,31]. In our study, we evaluated ROMO1 in gastrointestinal cancers in terms of mRNA and protein expression, clinical outcomes, and immune cells using various databases. ROMO1 expression was observed to differ among cancer types and to have high expression in gastrointestinal cancers (Figure 1). Particularly, a high expression of ROMO1 was associated with poor prognosis in LICH. Studies in the literature have shown that ROMO1 expression is increased in cancer types such as bladder [32], glioblastoma [33], prostate [28], and gastric cancer [8]. Jo et al. reported that ROMO1 protein is increased in colorectal cancer patients and may serve as a diagnostic marker [13]. Another study has shown that ROMO1 and the NF-κB pathway may regulate oxidative stress-induced tumor cell invasion in hepatocellular carcinoma [34].

In the present study, the ROMO1 methylation level in GI cancers tumors and associated normal tissues was analyzed using the UALCAN database. In addition, our findings show a substantial correlation between rising GI malignancies and rising ROMO1 expression. As a result, it appears that the methylation status is not the primary cause of the variations in ROMO1 complex gene expression. Thus, our results suggest that methylation level and GI tumors both play a significant role in controlling the expression of the ROMO1 complex gene. In all GI cancer types under investigation, these mechanisms result in alterations in gene expression, and anomalies may be directly linked to clinicopathological characteristics that may be applied in clinical treatment.

ROMO1 survival and predictive value in COAD, ESCA, LIHC, PAAD, and STAD were assessed using OS and DFS. The survival analysis results with GEPIA tools showed that high expression levels of ROMO1 did not correlate with a poor prognosis concerning COAD, ESCA, PAAD, and STAD (Figure 6). In particular, upregulation of ROMO1 was associated with poor prognosis in LIHC. ROMO1 may be a potential target for the diagnosis and treatment of LIHC. These findings also showed that ROMO1 might have distinct functions in GI cancers. Also, these results suggest that the ROMO1 gene may play a role in the overall survival of the analyzed GI cancer patients; more specifically, their increased expression was associated with a shorter overall survival. This association was confirmed by Kim et al. in non-small cell lung cancer, where favorable OS was found to be significantly associated with ROMO1 expression [35]. This was shown to be the main reason for the unfavorable survival rates due to elevated ROMO1-associated lymphatic metastasis. More research is required to elucidate the mechanism and determine whether ROMO1 has a specific function in various cancer types.

The immune system plays a vital role in preventing and treating tumors [36]. Tumor immune infiltration analysis showed that ROMO1 expression in COAD, LIHC, PAAD, and STAD was associated with the infiltration of immune cells (B cells, CD4+ T cells, CD8+ T cells, neutrophils, macrophages, and dendritic cells) (Figure 7). Another study reported that ROMO1 expression in prostate cancer was also associated with immune cells infiltrating the tumor, leading to changes in the tumor microenvironment and further increasing tumor heterogeneity [28].

In the present study, the STRING tool showed that the protein products of the analyzed genes interact with proteins of various signaling pathways related to, ERK, TGF-β pathway, and NF-κB extracellular matrix (ECM) pathways. It has been reported that ROS produced by ROMO1 trigger various signaling agents such as ERK, the TGF-β pathway, NF-κB, and ECM proteins and epithelial mesenchymal transition (EMT) factors, leading to metastasis, proliferation, and invasion [12,14,37,38]. The protein/gene interactions associated with ROMO1 and its potential molecular regulatory mechanisms were examined. ROMO1 is closely related to the inner mitochondrial membrane proteins (TIMM) family, such as TIMM17A, TIMM9, and TIMM8A (Figure 8). In recent years, the translocase of the TIMM proteins has been implicated in the development of various types of human cancer [39].

In the near future, more research involving patient groups with COAD, ESCA, LICH, PAAD, and STAD will validate the findings of this study. Furthermore, by conducting functional tests on cell lines derived from these specific gastrointestinal cancers in vitro, it will be possible to assess how the ROMO1 gene and other variables, such as possible medications, affect the expression of these genes and the course of cancer development and progression. All of these will make it possible to ascertain the end results and confirm if the ROMO1 gene under analysis may be utilized as targets for customized treatment or as prognostic/predictive elements in clinical practice. However, the article has some limitations; the role of ROMO1 needs to be experimentally confirmed in vivo and in vitro. Also, larger sample sizes in cancer and healthy groups are needed. Another limitation of our study is the differences in the numbers of healthy and cancer groups. Therefore, our study is limited due to limited sample sizes. A large number of experiments are needed to confirm the exact expression of ROMO1 gene in GI cancers and survival results. In addition, sample sizes should be similar between groups.

## 5. Conclusions

ROMO1 is significantly upregulated in gastrointestinal cancers and closely associated with clinicopathological features, which may play an essential role in the development of these cancers. Moreover, ROMO1 expression was closely associated with immune infiltration and may play a role in the regulation of tumor immunity. The bioinformatics analysis results suggest that the upregulation of ROMO1 may be linked to the development and progression of selected gastrointestinal cancers and may be associated with a faster progression of these cancers. The ROMO1 gene was found to have differential amplification in GI cancers (COAD, ESCA, LICH, PAAD, and STAD). On the other hand, the potential of the investigated ROMO1 gene could be expanded to include various signaling pathways during the carcinogenesis process. STRING analysis showed that this gene is present with various biological options such as cell survival, apoptosis, characteristic response, and ERK, TGF-β, and NF-κB signaling pathways. Despite the need for more experimental and clinical verification, some molecules within this network have the potential to function as disease diagnostic and treatment biomarkers. This might reveal fresh information about the molecular causes of GI cancers and possible treatment targets. In conclusion, the ROMO1 gene plays a significant part in the onset and spread of a few types of gastrointestinal malignancies; these tumors may be valuable in clinical practice as therapeutic targets and possible prognostic indicators.

## Data Availability

The data supporting this study’s findings are available on request from the corresponding author.
